# *Streptococcus pneumoniae* colonization associates with impaired adaptive immune responses against SARS-CoV-2

**DOI:** 10.1172/JCI157124

**Published:** 2022-04-01

**Authors:** Elena Mitsi, Jesús Reiné, Britta C. Urban, Carla Solórzano, Elissavet Nikolaou, Angela D. Hyder-Wright, Sherin Pojar, Ashleigh Howard, Lisa Hitchins, Sharon Glynn, Madlen C. Farrar, Konstantinos Liatsikos, Andrea M. Collins, Naomi F. Walker, Helen C. Hill, Esther L. German, Katerina S. Cheliotis, Rachel L. Byrne, Christopher T. Williams, Ana I. Cubas-Atienzar, Tom E. Fletcher, Emily R. Adams, Simon J. Draper, David Pulido, Rohini Beavon, Christian Theilacker, Elizabeth Begier, Luis Jodar, Bradford D. Gessner, Daniela M. Ferreira

**Affiliations:** 1 Clinical Sciences, Liverpool School of Tropical Medicine, Liverpool, United Kingdom.; 2 Liverpool University Hospitals National Health Service (NHS) Foundation Trust, Liverpool, United Kingdom.; 3 Tropical Disease Biology, Liverpool School of Tropical Medicine, Liverpool, United Kingdom.; 4 Jenner Institute, University of Oxford, Oxford, United Kingdom.; 5 Pfizer Vaccines, Collegeville, Pennsylvania, USA.

**Keywords:** Immunology, Virology, Adaptive immunity, Bacterial infections, T cells

## Abstract

**Background:**

Although recent epidemiological data suggest that pneumococci may contribute to the risk of SARS-CoV-2 disease, cases of coinfection with *Streptococcus pneumoniae* in patients with coronavirus disease 2019 (COVID-19) during hospitalization have been reported infrequently. This apparent contradiction may be explained by interactions of severe acute respiratory syndrome coronavirus 2 (SARS-CoV-2) and pneumococci in the upper airway, resulting in the escape of SARS-CoV-2 from protective host immune responses.

**Methods:**

Here, we investigated the relationship of these 2 respiratory pathogens in 2 distinct cohorts of health care workers with asymptomatic or mildly symptomatic SARS-CoV-2 infection identified by systematic screening and patients with moderate to severe disease who presented to the hospital. We assessed the effect of coinfection on host antibody, cellular, and inflammatory responses to the virus.

**Results:**

In both cohorts, pneumococcal colonization was associated with diminished antiviral immune responses, which primarily affected mucosal IgA levels among individuals with mild or asymptomatic infection and cellular memory responses in infected patients.

**Conclusion:**

Our findings suggest that *S*. *pneumoniae* impair host immunity to SARS-CoV-2 and raise the question of whether pneumococcal carriage also enables immune escape of other respiratory viruses and facilitates reinfection.

**Trial registration:**

ISRCTN89159899 (FASTER study) and ClinicalTrials.gov NCT03502291 (LAIV study).

## Graphical abstract

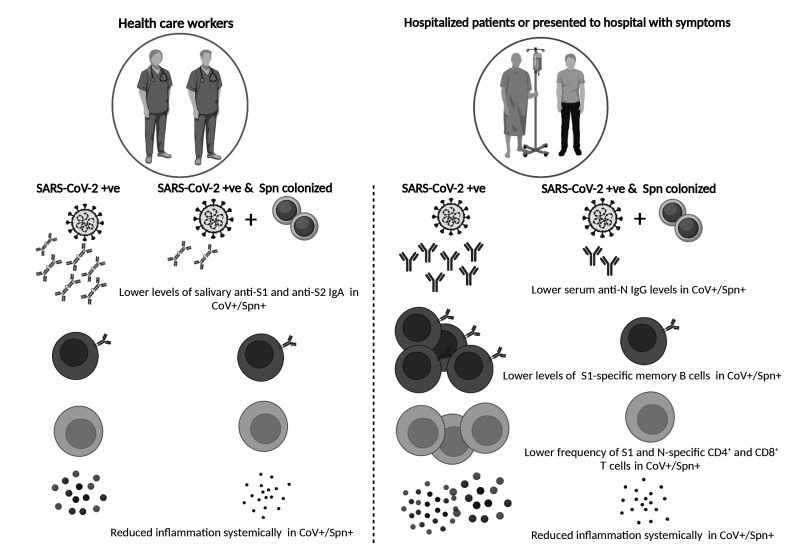

## Introduction

Despite the widespread global effects of the coronavirus disease 2019 (COVID-19) pandemic, few reports have assessed potential interactions between upper airway bacterial colonization and severe acute respiratory syndrome coronavirus 2 (SARS-CoV-2). Consequently, the contribution of respiratory bacterial pathogens to SARS-CoV-2 infection and pathogenesis remains poorly understood (1, 2). Post hoc analysis of 2 randomized clinical trials found that individuals vaccinated with pneumococcal conjugate vaccines (PCVs) showed a reduction of 30%–35% in hospitalizations for the endemic human coronaviruses (HCoV, OC43, and HKU1) associated with pneumonia in adults (3, 4) and lower respiratory infection in children (5). A recent observational study reported that the 13-valent pneumococcal conjugate vaccine (PCV13) in older adults was associated with a reduction of approximately 30% in COVID-19 disease, hospitalization, and death (6). Also, a recent epidemiological study reported higher mortality rates among patients with SARS-CoV-2 coinfection or subsequent infection (although rare events) within 28 days of invasive pneumococcal disease (IPD) in the United Kingdom (7).

Traditionally, virus-pneumococcus interaction in the upper airway has been thought to increase the risk of secondary pneumococcal pneumonia, particularly during influenza and respiratory syncytial virus (RSV) seasonal outbreaks (8). However, a substantially low proportion of patients with COVID-19 have had documented pneumococcal pneumonia based on culturing of blood or sputum samples collected during hospitalization, with specimen collection often occurring after the provision of antibiotics (1, 7, 9, 10). Bacterium-virus interaction in the upper airways could act synergistically to promote viral evasion by direct and indirect mechanisms (11, 12). Adaptive immune mechanisms play a critical role in protecting against viral infection, including against SARS-CoV-2 (13–15). *Streptococcus* *pneumoniae* (Spn) has been shown to have a modulatory effect on the antiviral immune responses mounted by the host, and the sequence of pathogen exposure in coinfection cases may also alter the disease outcome. Mice exposed to Spn prior to influenza A exhibited reduced antiviral serum IgG levels 1 month after infection (16), whereas a randomized, controlled human study of experimental pneumococcus/influenza coinfection reported diminished mucosal IgA responses to influenza antigens associated with pneumococcal carriage (17), resembling findings of the current study. Nonpneumococcus-specific cleavage of mucosal IgA1 by pneumococcal IgA1 proteases (18) could be a potential mechanism that contributed to the antiviral IgA reductions identified in both studies.

To study interactions of pneumococcus and SARS-CoV-2 and the effect of pneumococcus on host antiviral immune responses, we longitudinally sampled a cohort of health care workers (HCWs) at high risk for SARS-CoV-2 infection and patients with suspected COVID-19 disease. In both cohorts, we studied the prevalence of SARS-CoV-2 and pneumococcal colonization, associations of coinfection, and disease severity and evaluated immune responses and inflammation levels in the context of SARS-CoV-2 monoinfection and coinfection with pneumococcus. Last, we sought to assess whether pneumococcal carriage associated with a reduction in mucosal IgA to respiratory viruses could be due the activity of pneumococcus IgA1 protease as well as whether the order of infection of virus and pneumococcus was important. To answer these questions, we evaluated samples from our previous studies of live, attenuated influenza virus vaccine (LAIV) and pneumococcus coinfections (12, 17, 19).

## Results

### SARS-CoV-2 and Spn prevalence in the HCW and patient cohorts.

**Figure 1:**
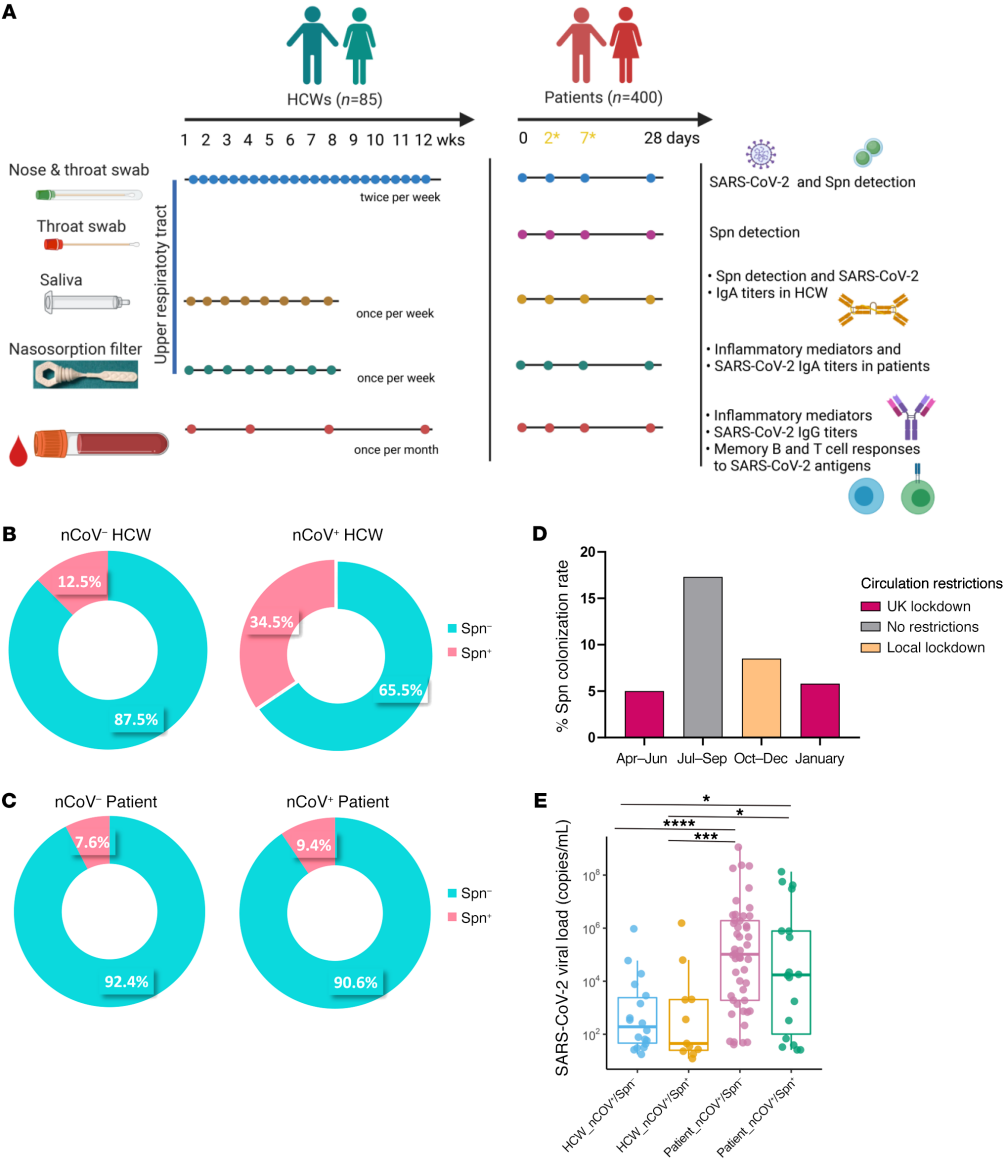
Prevalence of pneumococcal colonization among SARS-CoV-2–positive and –negative HCWs and patients. (**A**) Experimental design of the study with the sample type, sample collection schedule, and measurable-per-sample type depicted for both the HCW and patient cohorts. In the patient cohort, day 2 and day 7 samples were collected only for individuals who were hospitalized. (**B** and **C**) Doughnut charts showing the percentage of pneumococcal prevalence in (**B**) HCWs (*n =* 85) and (**C**) patients (*n =* 400) infected or noninfected with SARS-CoV-2. Fisher’s exact test was used to compare percentages. (**D**) Percentage of the pneumococcal colonization rate detected in the patient cohort during calendar periods with different circulation restrictions: 5% (6 of 119) from April to June; 17.3% (13 of 75) from July to September; 8.5% (13 of 154) from October to December; and 5.8% (3 of 52) in January. (**E**) Viral load levels expressed as RNA copies/mL, as detected by genesig RT-qPCR in noncolonized (*n =* 19, light blue) and Spn-colonized (*n =* 10, yellow) HCWs and noncolonized (*n =* 73, lilac) and Spn-colonized (*n =* 19, green) patients. *P < 0.05, ***P < 0.001, and ****P < 0.0001, by Kruskal-Wallis test for comparisons between groups.

We assessed the impact of pneumococcal carriage on SARS-CoV-2 viral replication and clinical outcomes in a cohort of frontline HCWs (*n =* 85, median age: 35 years; IQR: 27.5–46.5) and a cohort of patients who presented to the hospital with suspected COVID-19 disease (*n =* 400 patients; median age: 61 years; IQR: 48–72). Participants were screened for both SARS-CoV-2 and Spn presence in the naso/oropharynx (Figure 1A). Among HCWs, 34% (29 of 85) tested positive for SARS-CoV-2 at any time point during the 3-month follow-up period of the study on a combined nose and throat (NT) swab or on NT swab and saliva sample, and all of these HCWs experienced asymptomatic or mildly symptomatic viral infection (20). In the patient cohort, 63.5% (255 of 400) tested positive for SARS-CoV-2 at the time of recruitment to the study on the basis of NT swabs, and their symptoms ranged from moderate to severe (21).

In the HCW cohort, the overall pneumococcal colonization rate was 20% (17 of 85). We observed an increased pneumococcal prevalence in SARS-CoV-2–positive participants compared with SARS-CoV-2–negative participants (34.5% [10 of 29] vs. 12.5% [7 of 56], respectively, *P =* 0.023) (Figure 1B), with 7 of 10 individuals acquiring SARS-CoV-2 while already being colonized with Spn, and 3 of 10 having a concurrent infection (Supplemental Figure 1; supplemental material available online with this article; https://doi.org/10.1172/JCI157124DS1). In the patient cohort, the overall Spn colonization rate was 8.5% (35 of 400), and the prevalence of Spn colonization did not differ among the SARS-CoV-2–positive and SARS-CoV-2–negative individuals (9.4% [24 of 255] vs. 7.6% [11 of 145], respectively) (Figure 1C). In SARS-CoV-2–infected patients, pneumococcal colonization did not associate with increased disease severity, as defined by the NIH severity score (median: 4, IQR: 3–4 in both groups) (Table 1) or reduced survival rates (Supplemental Figure 2). Also, these 2 groups did not differ with regard to the days of sample collection after symptoms onset (median: 41 days, IQR: 29–57 vs. 47 days, IQR: 36–59) (Table 1). However, recruitment of patients who had already developed symptoms made the order of pathogen infection unknown. During the 9-month recruitment period for the patient study, we observed fluctuations in pneumococcal carriage rates, with lower colonization rates during periods of local and UK lockdowns (Figure 1D).

Although, the SARS-CoV-2 upper airway viral load did not differ significantly according to Spn carriage status in either cohort, the HCW noncolonized group had a 4.4-fold higher median value (median: 2.01 × 10^2^ RNA copies/mL, IQR: 4.02 × 10^1^ to 4.03 × 10^3^) than did the Spn-colonized group (4.5 × 10^1^ RNA copies/mL, IQR: 2.30 × 10^1^ to 2.03 × 10^3^ in Spn^+^ individuals). Similarly, in the patient cohort, the noncolonized group had a 6-fold higher viral load (median: 1.04 × 10^5^ RNA copies/mL, IQR: 1.89 × 10^3^ to 1.94 × 10^6^) than did the Spn-colonized group (1.74 × 10^4^ RNA copies/mL, IQR: 6.12 × 10^1^ to 8.14 × 10^6^ in Spn^+^ individuals). The patient cohort (both Spn-colonized and noncolonized groups) had a higher viral load compared with that in the HCW cohort (Figure 1E).

### Impaired mucosal antibody responses to SARS-CoV-2 in pneumococcus-colonized individuals.

IgA plays a crucial role in the antiviral immune defense of mucosal surfaces (14, 22, 23). In this study, the levels of mucosal IgA to surface SARS-CoV-2 antigens, such as receptor binding domain (RBD), spike protein subunit 1 (S1) and subunit 2 (S2), and the internal nucleocapsid protein (N) were measured in saliva samples from HCWs and nasal lining fluid from individuals in the patient cohort (due to difficulties in acquiring saliva from patients) 1 month after SARS-CoV-2 infection.

**Figure 2:**
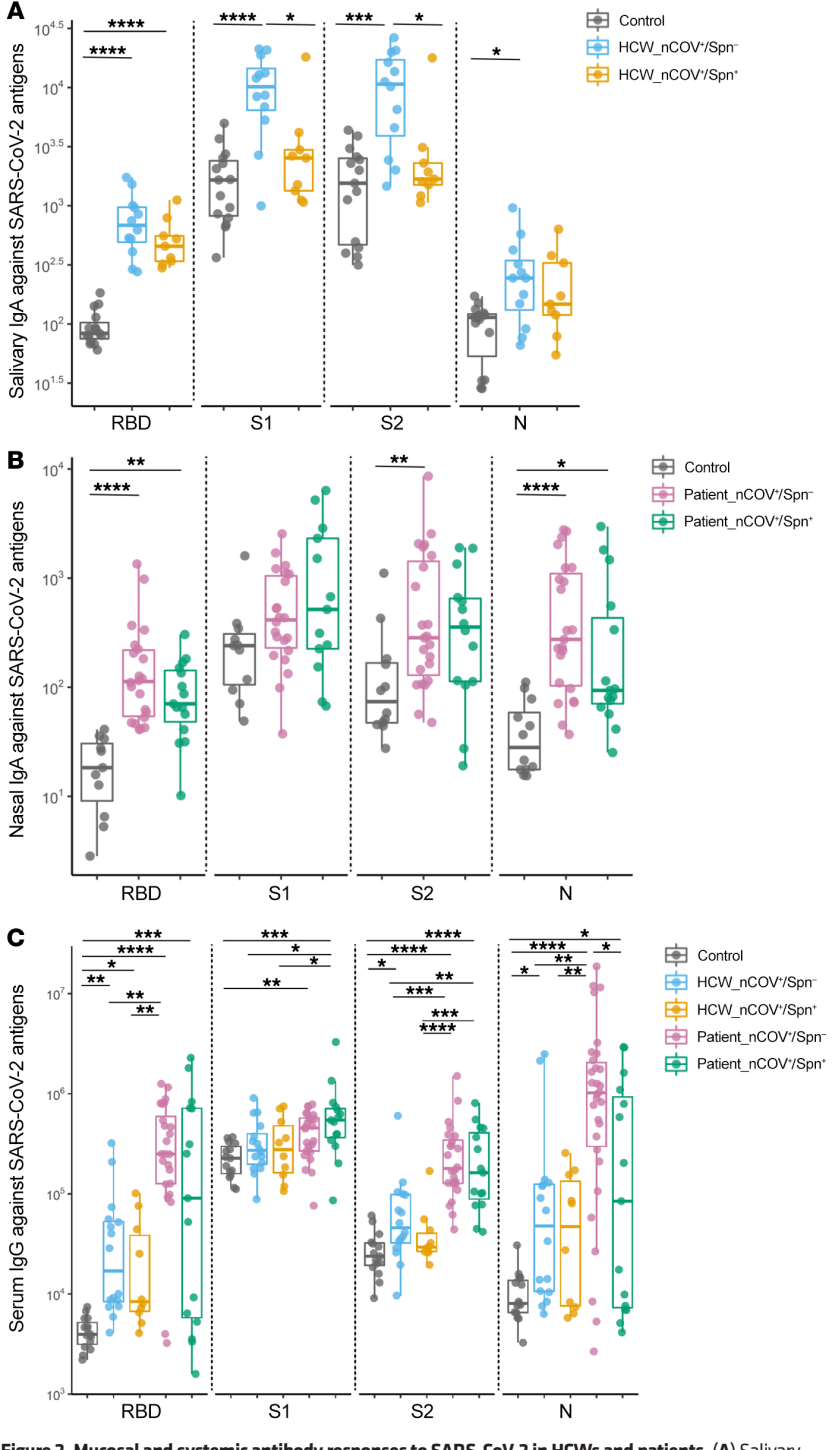
Mucosal and systemic antibody responses to SARS-CoV-2 in HCWs and patients. (**A**) Salivary IgA titers against SARS-CoV-2 RBD, S1, S2, and N proteins in HCWs, divided into noncolonized (*n =* 12) and Spn-colonized (*n =* 9) groups and in unexposed, healthy controls (*n =* 15). (**B**) Nasal IgA titers against SARS-CoV-2 RBD, S1, S2, and N proteins in patients, divided into noncolonized (*n =* 23) and Spn-colonized (*n =* 15) groups and in unexposed, healthy controls (collected before 2019, *n =* 12). (**C**) Serum IgG titers in HCWs (*n =* 16 noncolonized and *n =* 10 Spn-colonized), patients (*n =* 24 noncolonized and *n =* 14 Spn-colonized), and unexposed, healthy controls (*n =* 15). Both mucosal and serum antibody titers from SARS-CoV-2–positive participants were measured during the convalescent phase of the viral infection. Antibody levels are expressed as AU. Medians with IQRs are shown for antiviral responses. *P < 0.05, **P < 0.01, ***P < 0.001, and ****P < 0.0001, by Kruskal-Wallis test for comparisons between groups.

In the HCW cohort, among SARS-CoV-2–positive participants, noncolonized HCWs had higher salivary IgA levels than did Spn-colonized HCWs for all SARS-CoV-2 antigens assessed, with statistically significant differences for S1 and S2 between the 2 HCW groups (median 4.1- and 6.4-fold change of IgA to S1 and S2, respectively; *P =* 0.035 and *P =* 0.028, respectively) (Figure 2A).

Among SARS-CoV-2–positive patients, there was a trend of overall weakened IgA induction against SARS-CoV-2 antigens in the Spn-colonized versus the noncolonized group, however, we found no significant differences between the 2 groups (Figure 2B). Noncolonized individuals mounted robust nasal IgA against SARS-CoV-2 antigens (RBD, S2, and N), with the exception of S1, for which titers did not differ significantly from those of the control group. This group had the highest antibody fold increase against RBD and N (6.2-fold and 9.4-fold increase from control levels; *P <* 0.0001 in both) (Figure 2B). Spn-colonized participants showed a lesser increase against RBD and N (2.2- and 3.2-fold increase from control levels; *P =* 0.002 and *P =* 0.014, respectively) (Figure 2B) and a moderate induction of nasal IgA against S1 and S2, with titers that were not significantly higher from those of the control group (Figure 2B).

We measured antiviral IgG responses in convalescent sera from both cohorts. In the HCW cohort, we noted a trend of overall lower levels of IgG induction for RBD and S2 antigens in the Spn-colonized participants compared with noncolonized participants (Figure 2C). Noncolonized participants showed a moderate rise in IgG titers against all SARS-CoV-2 proteins, except S1, whereas the Spn-colonized counterparts mounted lower IgG responses against those viral antigens, with only anti–RBD IgG levels differing significantly from those detected in healthy controls. (Figure 2C). In the patient cohort, IgG levels against N protein were greater in the noncolonized patients when compared with those mounted by the Spn-colonized counterparts (median 12-fold difference, *P =* 0.014) and 2-fold higher against RBD in the noncolonized versus the Spn-colonized group (*P =* 0.10) (Figure 2C). The 2 patient groups raised similar IgG levels against the spike subunits. In agreement with findings that disease severity correlates with increased levels of systemic IgG against SARS-CoV-2 (24), we observed that IgG titers against viral antigens were consistently higher in patients than in HCWs (Figure 2C).

### Experimentally induced pneumococcal colonization impairs nasal IgA against influenza antigens but only when colonization precedes viral infection.

We have previously observed reduced mucosal IgA, but not IgG and IgM, responses to influenza antigens in Spn-colonized individuals when colonized with serotype 6B three days before administration of LAIV (17). Pneumococcal IgA1 protease is a cell-associated enzyme that cleaves human IgA1, but not IgA2 (18). To test the involvement of IgA1 in the reduction of antiviral mucosal IgA, we evaluated the association of pneumococcal carriage with virus-specific IgA1 versus IgA2 levels in nasal mucosa samples previously collected in 2 LAIV-pneumococcal coinfection studies of individuals with known onset of viral and Spn infection (12, 19).

Influenza-specific IgA, IgA1, and IgA2 levels were measured in nasal wash samples at baseline and on day 24 after LAIV administration for a subset of participants (*n* = 15 Spn^+^ and *n* = 15 Spn^–^) who received LAIV 3 days after pneumococcal challenge. While no to little induction of total IgA and IgA subclasses against influenza antigens was observed from baseline levels in Spn-colonized participants, the noncolonized group exhibited a median 3-, 1.9-, and 1.7-fold increase in influenza-specific IgA, IgA1, and IgA2 titers, respectively (Supplemental Figure 3A). When the order of infection was inverted (LAIV infection occurred 3 days before pneumococcal challenge) (19), the levels of influenza-specific IgA did not differ between Spn-colonized and noncolonized participants (IgA median 1.5- and 1.6-fold increase, respectively; *P =* 0.28) (Supplemental Figure 3B). Given the insufficient nasal material from the current study cohorts, we could not assess the IgA subclasses in those samples.

### Spn colonization is associated with decreased levels of memory B cells against SARS-CoV-2.

**Figure 3:**
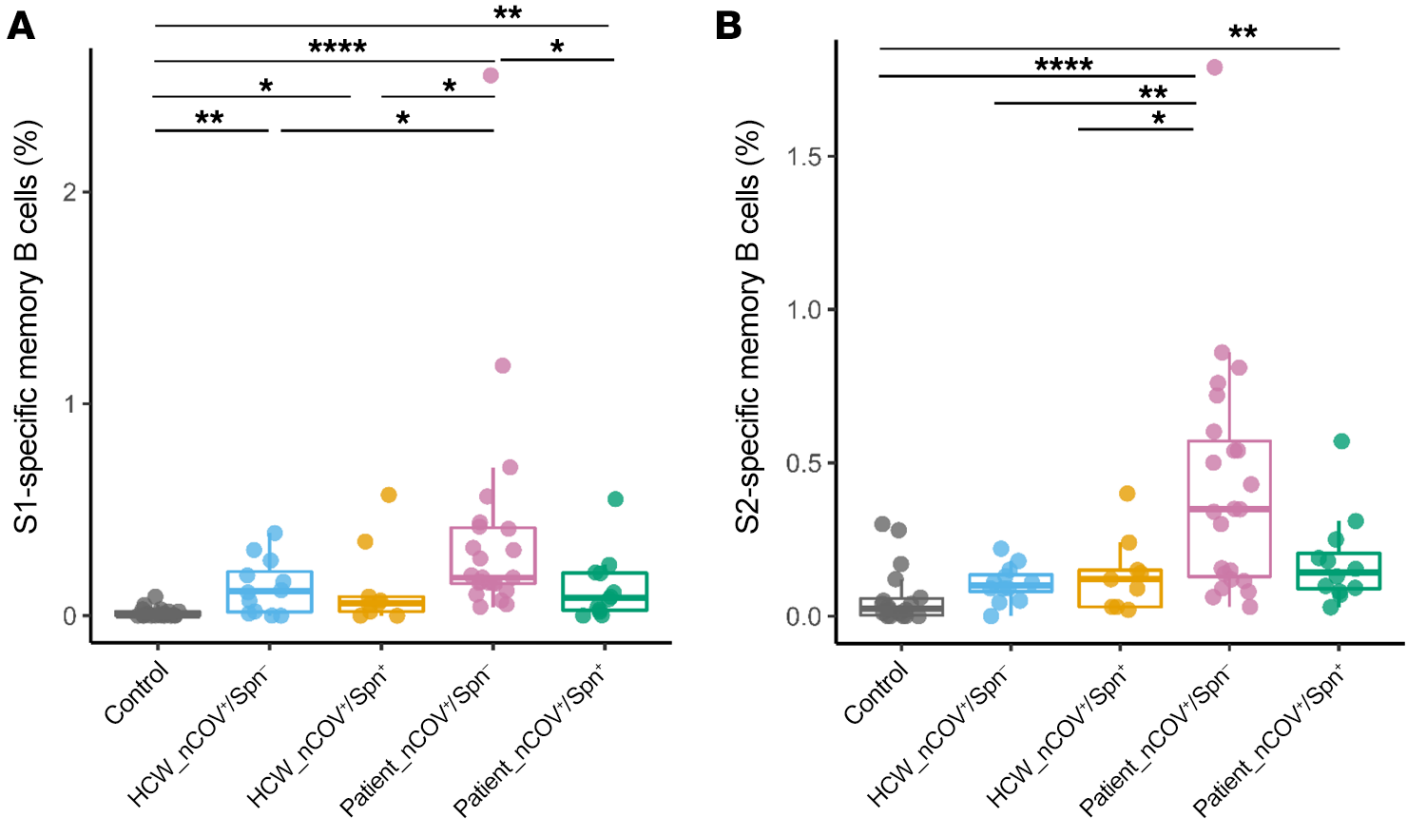
SARS-CoV-2–specific memory B cells in HCWs and patients. Percentage of (**A**) S1- and (**B**) S2-specific memory B cells within CD19^+^CD27^+^ memory B cells in HCWs (*n =* 12 noncolonized and *n =* 9 Spn-colonized), recovered patients (*n =* 23 noncolonized and *n =* 12 Spn-colonized), and healthy controls (*n =* 18). Medians with IQRs are shown, and each dot represents an individual. *P < 0.05, **P < 0.01, and ****P < 0.0001, by Kruskal-Wallis test.

Memory B cells are of great importance for long-term humoral immunity. To identify SARS-CoV-2–specific memory B cells, we used fluorescently labeled S1 and S2 antigens in PBMCs from HCWs and recovered patients (Supplemental Figure 4). Overall, Spn-colonized participants showed a trend of reduced frequency of memory B cells against SARS-CoV-2 antigens compared with their noncolonized counterparts, which we observed in both HCW and patient cohorts (Figure 3). In the HCW cohort, the proportion of S1-specific memory B cells was significantly higher in healthy controls in the noncolonized (0.12% vs. 0.01%, *P =* 0.003) and Spn-colonized (and 0.06% vs. 0.01%, *P =* 0.031) groups but less pronounced in the latter group (Figure 3A). In the patient group, noncolonized individuals had the highest frequencies of S1- and S2-specific memory B cells when compared with Spn-colonized patients and any other group. Specifically, noncolonized patients had a greater proportion of S1-specific memory B cells (0.18% vs. 0.08%, *P =* 0.027) and a strong trend toward a higher S2-specific memory B cell proportion than did the Spn-colonized counterparts (0.35% vs. 0.14%, *P =* 0.09) (Figure 3B).

### Lack of SARS-CoV-2–specific T cell responses in patients colonized with Spn.

**Figure 4:**
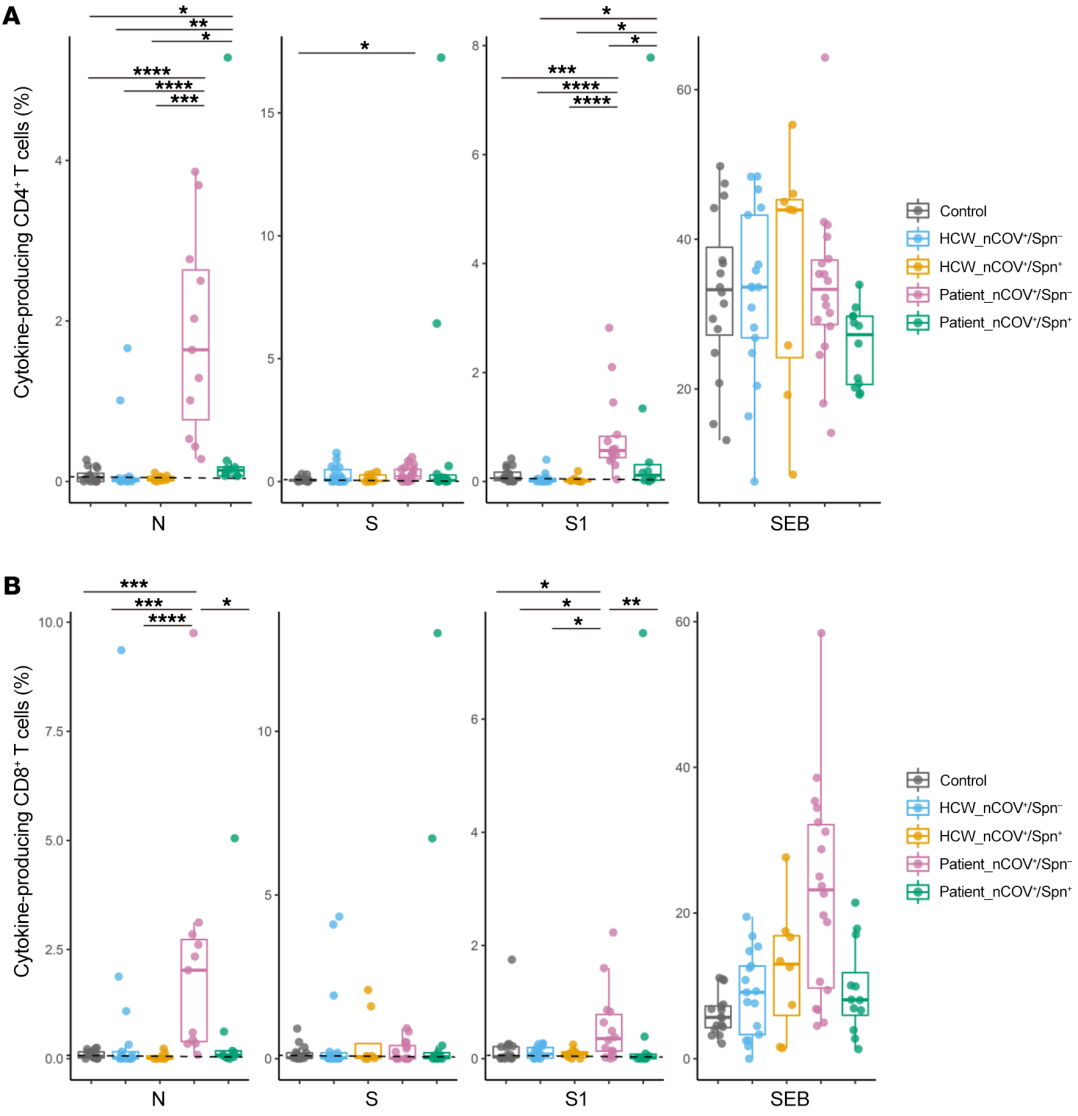
SARS-CoV-2–specific T cell responses in HCWs and patients. Percentage of (**A**) cytokine-producing (IFN-γ, TNF-α, IL-2) CD4^+^ and (**B**) CD8^+^ T cells after ex vivo PBMC stimulation with N, S1, and S peptide pools in SARS-CoV-2–positive HCWs (*n =* 17 noncolonized and *n =* 8 Spn-colonized), recovered patients (*n =* 17 colonized and *n =* 14 Spn-colonized), and healthy controls (*n =* 16). One peptide pool was used per condition. SEB was used as a positive control and DMSO as the negative control (nonstimulated cell condition – mock). Background (mock) was subtracted from the peptide-stimulated conditions to remove nonspecific signals. Data indicate positivity for any of the 3 measured cytokines. Medians with IQRs are shown, and each dot represents an individual. *P < 0.05, **P < 0.01, ***P < 0.001, and ****P < 0.0001, by Kruskal-Wallis test.

To assess CD4^+^ and CD8^+^ T cell–mediated recall responses (Supplemental Figure 5) in HCWs and patients, as well as in healthy uninfected controls, PBMCs were stimulated ex vivo with N-, S-, and S1-defined peptide pools from SARS-CoV-2. In the HCW cohort, including both Spn-colonized and noncolonized individuals, overall CD4^+^ T cells responses did not differ significantly from those of healthy controls (Figure 4A). In the patient cohort, the magnitudes of T cell responses to N, S1, and S were greater in the noncolonized group than in the Spn-colonized group and were one of the highest in either study cohort. Median percentages of specific CD4^+^ T cells for N, S1, and S were 1.64% (IQR: 0.53–2.77), 0.22% (IQR: 0.08–0.54), and 0.57% (IQR: 0.41–1.09), respectively, in the noncolonized group versus 0.14% (IQR: 0.07–0.22), 0.08% (IQR: 0.018–0.38), and 0.12% (IQR: 0.02–0.60), respectively, in the Spn-colonized group. IL-2 was the most abundantly produced cytokine, and similar secretion patterns were observed for TNF-α and IFN-γ, as described above. The cytokine-specific (IFN-γ, ΤNF-α, and IL-2) CD4^+^ T cells responses to each peptide per group are shown in Supplemental Figure 6.

We also assessed the SARS-CoV-2 N-, S-, and S1-specific CD8^+^ T cell responses in the same samples. Similar to CD4^+^ T cell responses, both the number of responders and magnitudes of CD8^+^ responses to N and S1 were the highest and most robust, respectively, in the noncolonized patients. In this group, the median CD8^+^ T cell responses for N and S1 were 2.03% (IQR: 0.35%–2.85%) and 0.36% (IQR: 0.11%–0.84%), respectively (Figure 4B). CD8^+^ T cell responses to the S peptide pool were close to the lower limit of detection (LOD) in all groups. CD8^+^ responses to N and S1 were impaired in the Spn-colonized patients and differed significantly from those of their noncolonized counterparts (median: 0.08% vs. 2.03%, *P =* 0.019 and median: 0.025% vs. 0.36%, *P =* 0.009, respectively) (Figure 4B).

### Distinct nasal inflammation profile between HCWs and patients with COVID-19.

**Figure 5:**
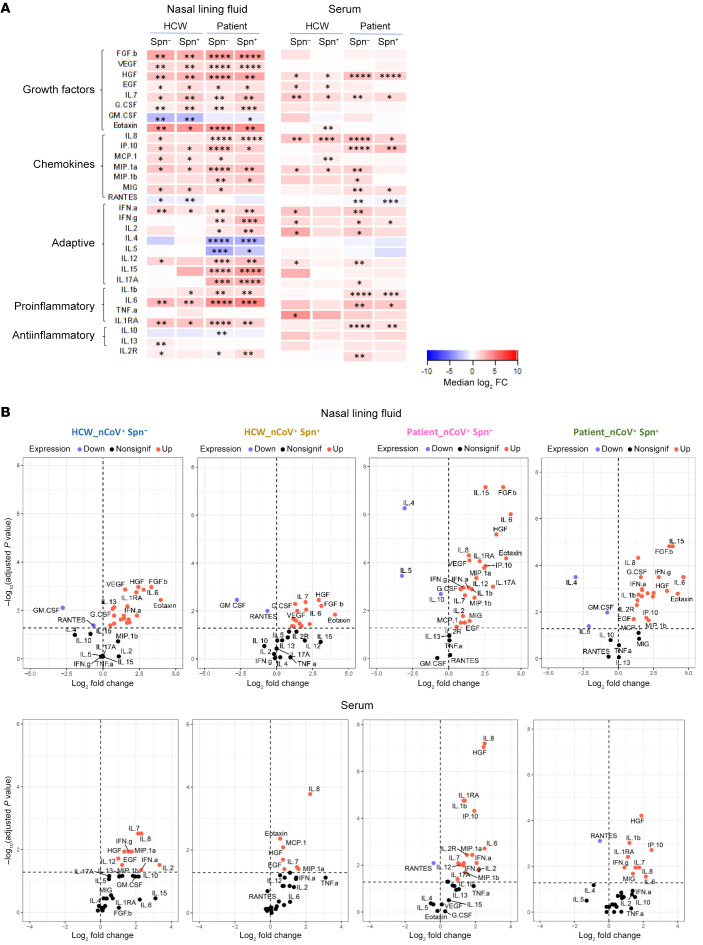
Cytokine concentrations in nasal lining fluid and serum. (**A**) Heatmaps showing the median log_2_ fold change (FC) in the levels of 30 cytokines in nasal lining fluid and serum of noncolonized and Spn-colonized HCWs and patients during the acute phase of SARS-CoV-2 infection versus unexposed healthy controls. Upregulation (red) and downregulation (blue) are shown compared with cytokine levels in the control group. Cytokines were clustered in active cytokine families. (**B**) Volcano plots showing the median log_2_ fold change versus healthy controls (*n =* 17) per cytokine in nasal lining fluid and serum of noncolonized HCWs (*n =* 17), Spn-colonized HCWs (*n =* 9), noncolonized patients (*n =* 70), and Spn-colonized patients (*n =* 19). The horizontal dotted line represents the cutoff of significance (adjusted *P* = 0.05, after FDR correction of the *P* value), whereas the vertical dotted line represents a cutoff point for determining whether the levels of cytokines were higher (right, red) or lower (left, blue) compared with those of the healthy control group. Statistical comparisons were applied between each study group and the healthy control group using the Mann-Whitney *U* test, followed by Benjamini-Hochberg correction for multiple testing. Nonsignif, nonsignificant; Down, downregulation; Up, upregulation.

SARS-CoV-2–induced nasal and systemic inflammatory responses were assessed by measuring the levels of 30 cytokines in the nasal fluid and serum during the early phase of the viral infection in the HCW and patient cohorts. In the nose, the HCW groups showed a lack of upregulation of cytokines that functionally promote T and B cell maturation and differentiation (Figure 5A). In blood, noncolonized patients exhibited an increased inflammatory profile (16 of 30 cytokines were upregulated) compared with that of their Spn-colonized counterparts (upregulation of 9 of 30 cytokines) (Figure 5A). It is important to note that the levels of IL-2 and IL-12, which are key cytokines for T cell proliferation and activation, did not differ from that in the control in both the HCW and patient Spn-colonized groups (Figure 5A). The levels of nasal and serum cytokines in each group were also plotted on the basis of significance and fold-change difference compared with the control group (Figure 5B).

**Figure 6:**
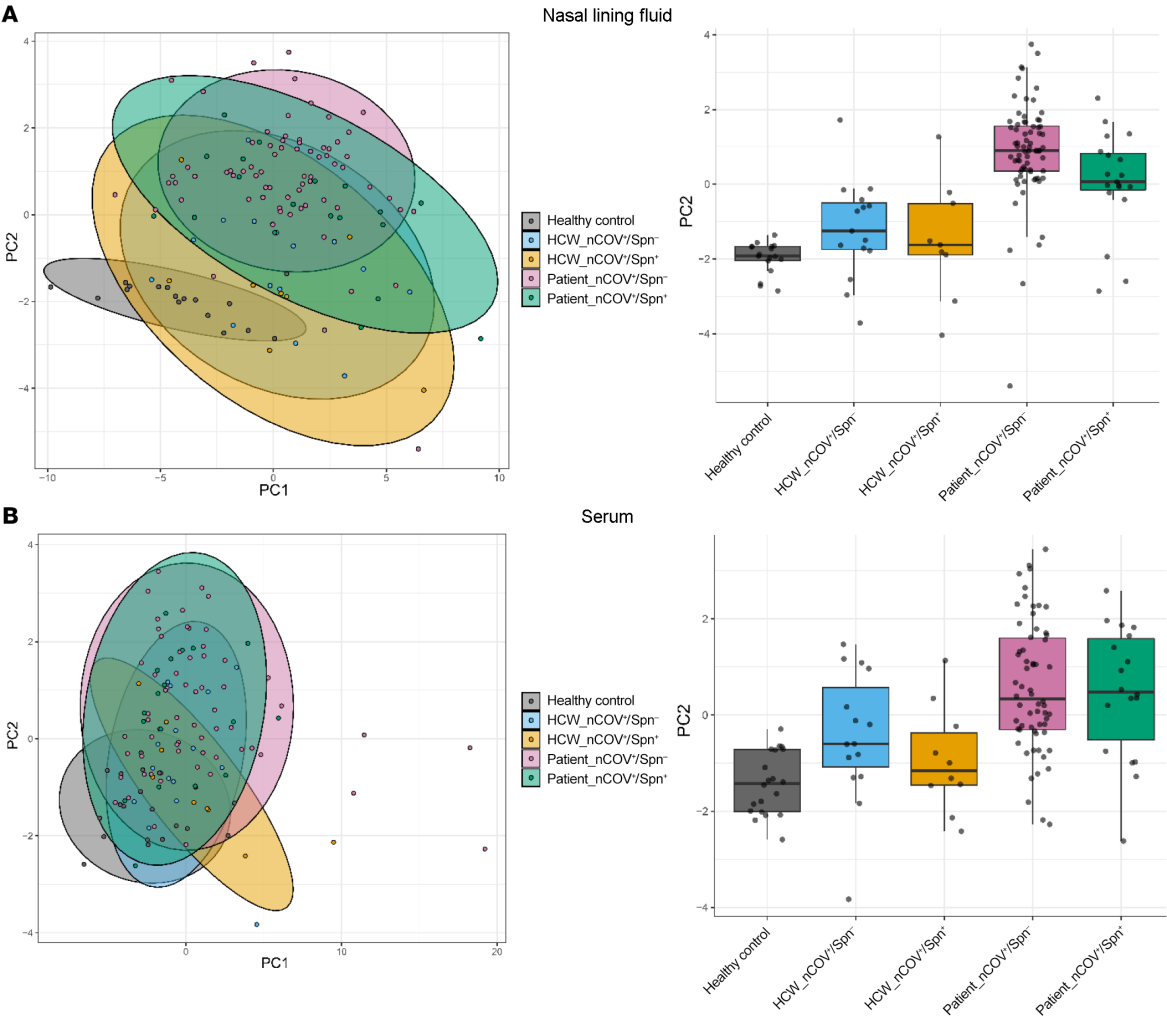
Nasal and serum inflammatory profiles of SARS-CoV-2 infection and coinfection with Spn in HCWs and patients. PCA of 30 cytokines in (**A**) nasal lining fluid and (**B**) serum of healthy controls (gray), noncolonized HCWs (light blue), Spn-colonized HCWs (yellow), noncolonized patients (lilac), and Spn-colonized patients (green). PC1, principal component 1; PC2, principal component 2.

We also performed an unsupervised analysis to assign profiles to each group. Principal component analysis (PCA) was applied on all analytes in nasal lining fluid (Figure 6A) and serum (Figure 6B) for all groups and the control. In the nose, patient groups were segregated together and away from the healthy control group in the second principal component (Figure 6A), with the noncolonized group having a more distinctive profile. The HCW groups had a similar inflammatory profile that clustered between the control and patient groups (Figure 6A). In the serum, the patient groups also segregated together and showed a very similar profile, which differed from that of the control group in the second principal component. The HCW groups clustered again between the control and patient groups, with the Spn-colonized HCWs appearing as a heterogeneous group (Figure 6B).

## Discussion

Here, we report the first immunological analysis to our knowledge of SARS-CoV-2 infection in the context of coinfection with pneumococcus among 2 distinct cohorts — asymptomatic and mildly symptomatic HCWs and patients who experienced moderate to severe symptoms during SARS-CoV-2 infection. More important, to our knowledge, this is the first comprehensive analysis showing the potential role of Spn carriage in modulating the host immune responses against SARS-CoV-2. Our findings have potential implications for other respiratory viruses.

Humoral and cellular antiviral immune responses varied substantially by pneumococcal carriage status both in HCW and patient cohorts, suggesting that colonization of upper airways by pneumococci affects host immunity to SARS-CoV-2. This effect was more apparent in the nasal mucosa of HCWs, where diminished salivary anti–S1 and anti–S2 IgA levels were observed in the pneumococcus-colonized individuals. In the patient cohort, we found that pneumococcal carriage was associated with reduced SARS-CoV-2–specific memory B cells and weakened T cell responses, particularly CD4^+^ T cell responses.

SARS-CoV-2 infection and virus replication start in the naso/oropharynx — the primary site of infection (25). Mucosal antibodies, particularly secretory IgA, play an important role in the defense against respiratory viruses (26, 27). For SARS-CoV-2 infection, in vitro studies with monoclonal anti–spike IgA demonstrated the superiority of IgA to block binding to the angiotensin-converting enzyme 2 (ACE2) receptor compared with the IgG isotype (28), and SARS-CoV-2 challenge studies in mice highlighted that mucosal anti–spike IgA production is critical for sterilizing immunity in the upper respiratory tract (27). We found that Spn-colonized HCWs had diminished IgA responses against SARS-CoV-2 S1 and S2 proteins compared with their noncolonized counterparts and that these responses did not differ between the Spn-colonized and noncolonized groups in the patient cohort. This may indicate that such an immune-suppressive effect is more important at the early phases of SARS-CoV-2 infection and less relevant once infection has progressed to symptomatic and severe disease.

We further investigated a potential mechanism responsible for the association between bacterial colonization and IgA-immunosuppressed responses against viral infections using an influenza virus/Spn coinfection model. We observed that the preceding pneumococcal colonization impaired the induction of both influenza-specific IgA1 and IgA2 nearly 1 month after influenza infection — an effect that was not seen in the absence of pneumococcal colonization. This suggests that cleavage of nonpneumococcus-specific IgA1 by the pneumococcal IgA1 protease most likely is not a mechanism through which pneumococcus contributes to the reduction in mucosal IgA against other pathogens. When influenza infection preceded experimentally induced pneumococcal colonization, both Spn-colonized and noncolonized, LAIV recipients mounted similar influenza-specific IgA levels. These findings imply that the order of exposure to respiratory pathogens during coinfection can affect some of the defense mechanisms. Differential nasal inflammatory responses during the early stages of infection, driven by either the virus or Spn*,* depending on the order of infection, may have a differential effect on downstream immune responses (12, 16, 17), altering the dynamics between the pathogens (19).

It has been shown that Spn stimulates IFN-I production and upregulates the expression of IFN-stimulated genes in both mouse and human studies (17, 29). Therefore, it is possible that pneumococcal colonization interferes with the replication cycle of the virus (30, 31) and contributes to host antiviral defenses by governing the production of IFNs (32, 33). Here, despite a trend of higher viral load in the noncolonized groups, we did not observe significant viral load differences between pneumococcus-colonized and noncolonized individuals. However, as the SARS-CoV-2 viral load changes rapidly from day to day, the nature of the study prohibited the assessment of such a time course–dependent variable (34, 35).

Consistent with published studies of COVID-19 infection (36, 37), we observed inflammatory responses, including IL-6, IP-10, IL-1b and IL-8, in nasal lining fluid and serum in both cohorts, with increased cytokine induction in the patient groups, particularly the noncolonized individuals. The induction of cytokines that influence T cell activation (IL-2, IL-12, IFN-γ, IL-15, IL-17A), which subsequently assists B cell maturation (38), was distinctive in the nasal mucosa of the patient cohort. Impairment of inflammatory response in the nasal mucosa could also affect the influx of effector immune cells and influence downstream immune responses (12, 17). Also, pneumococcus-colonized individuals in both cohorts exhibited a lack of IL-2 and IL-12 induction in the serum, which could partially explain the weakened T cell responses observed in those groups.

Consistent with previous studies, convalescent patients mounted higher serum IgG levels than did the asymptomatic and mildly symptomatic HCWs (24, 39), showed an increased frequency of memory B cells, broader and stronger T cell responses in the convalescent phase (40), and had elevated acute proinflammatory responses in both the nose and blood (41). Coordinated immunity by all 3 branches of adaptive immunity is more likely to protect against SARS-CoV-2 reinfection, as it is seen in protection against other infectious diseases (39), whereas suboptimal immunity against SARS-CoV-2 could allow reinfection to occur.

Our study has limitations. Diagnosis of coinfection was complex among the patients, as pneumococci might be carried by the patient before the viral infection or might be picked up later. High use of antibiotics (potentially prescribed at an outpatient visit) and reduced social mixing may have affected the prevalence and dynamics of transmission of other respiratory pathogens such as RSV (1, 42), and most likely pneumococcus. Hence, we observed a decreased prevalence of pneumococcus infection among patients — particularly during periods of national lockdown, which subsequently limited the number of SARS-CoV-2/ pneumococcus–coinfected individuals studied here. Thus, despite inclusion of nearly 500 participants in our study, we were able to evaluate the pneumococcus-mediated immunomodulatory effects only for a relatively small number of individuals, limiting the ability to do further stratifications. In addition, differences in age and underlying disease between the HCW and patient cohorts are factors that may have potentially affected the course and outcome of the disease. Further studies, ideally in the setting of a controlled human coinfection model, are needed to explore Spn–respiratory virus interactions and the biological mechanisms through which pneumococci assist viruses to subvert immune responses at the primary site of infection.

Despite the observational design, our study has identified pneumococcal colonization as a variable that can modulate host immune responses to SARS-CoV-2 infection, an effect that was observed in both cohorts despite the aforementioned differences. An impaired adaptive immunity against SARS-CoV-2 natural infection could potentially increase susceptibility to subsequent SARS-CoV-2 infection. The increased evidence of PCV-induced protection against lower respiratory infections associated with viral infection and the broader ability of pneumococci to interact with respiratory viruses in a way that increases pneumococcal virulence or viral pathogenicity or that impairs antiviral immune responses highlight the importance of PCVs in both pediatric and older adult populations as an additional public health tool for those who are at increased risk of pneumococcal and viral lower respiratory infections.

## Methods

Further information can be found in Supplemental Methods.

### Study design

This study combined participants recruited into 2 prospective cohort studies of (a) frontline HCWs (*n =* 85) and (b) patients (*n =* 400) who presented to the hospital. HCWs in a variety of roles were enrolled in the SARS-CoV-2 Acquisition in Frontline Healthcare Workers – Evaluation to inform Response (SAFER) study between March 30, 2020, and April 9, 2020, at the Royal Liverpool University Hospital (RLUH) in Liverpool, United Kingdom. Eligible HCWs (aged ≥18 years) were asymptomatic at the time of enrollment to the study. Screening against SARS-CoV-2 and Spn was performed on NT swabs and saliva samples (Figure 1A). Symptom reporting was done via a questionnaire completed twice weekly, accompanying each sampling episode (20).

For the patient cohort, adults (aged ≥18 years) who had signs and symptoms of suspected COVID-19 infection and were hospitalized at RLUH, Aintree University Hospital (Liverpool, United Kingdom), or Whiston Hospital (Merseyside, United Kingdom) between April 2020 and January 2021, were recruited into the Facilitating A SARS Cov-2 Test for Rapid Triage (FASTER) study, regardless of disease severity, race, ethnicity, sex, pregnancy or nursing status, or the presence of other medical conditions (Table 2). Screening against SARS-CoV-2 and Spn was performed on throat swabs, NT swabs, and saliva samples (Figure 1A).

### Bacterial DNA extraction and Spn quantitative PCR

Bacterial genomic DNA was extracted from both raw and culture-enriched (CE) material from throat swabs, NT swabs, and saliva samples for the patient cohort and from NT and saliva samples for the HCW cohort, as previously described (43). Briefly, bacterial DNA was extracted using the Agowa Mag Mini-DNA Extraction Kit (LGC Genomics). The presence of pneumococci was determined by sequential singleplex quantitative PCR (qPCR) targeting the *lyt*A (44) and *pia*B genes (45), using the QuantStudio 5 system (Thermo Fisher Scientific), as previously described (45). Briefly, 20 μL PCR mix consisted of 12.5 μL 1 × TaqMan Universal PCR Master Mix (Life Technologies, Thermo Fisher Scientific), 0.225μL or 0.2μL 100 μM each of the *lytA* or *piaB* primer, respectively, 0.125 μL or 0.175 μL 100 μM of the *lytA* or *piaB* probe, respectively, molecular grade water (Thermo Fisher Scientific), and 2.5 μL of the extracted DNA. Thermal cycling conditions were as follows: 10 minutes at 95°C and 40 cycles of 15 seconds at 95°C and 1 minute at 60°C. A negative DNA extraction control (parallel extraction from sample buffer only), a qPCR negative control (master mix only), a qPCR positive control (pneumococcal Spn15B strain), and duplicates of each sample were amplified. A standard curve of a 10-fold dilution series of genomic DNA extracted from TIGR4 was used. Samples were considered to be pneumococcus positive if both genes were present. All samples were assessed by a *lytA* qPCR, and those that were positive underwent a *piaB* qPCR. Samples were considered *lytA* positive if 1 or 2 yielded a Ct of fewer than 40 cycles. The threshold between plates was normalized according to the positive control Ct values.

### SARS-CoV-2 RNA extraction and reverse transcription qPCR

SARS-CoV-2 RNA was extracted from NT swabs in Amies solution or saliva, as previously described (46). Briefly, viral RNA was extracted using the QIAamp Viral RNA Mini Kit (QIAGEN), and 8 μL of extracted RNA was tested using the Real-Time Coronavirus COVID-19 PCR (genesig) assay. Virus copies/mL were quantified using the manufacturer’s positive control (1.67 × 10^5^ copies/μL) as a reference.

### Immunological analyses of SARS-CoV-2–positive participants

SARS-CoV-2–positive individuals from both cohorts (all Spn-colonized individuals and a subset of noncolonized HCWs and patients) were stratified by pneumococcal colonization status. Pre-pandemic samples from healthy, unexposed individuals were also included, resulting in 5 groups used for the immunological analysis: (a) HCWs nCOV^+^ Spn^–^; (b) HCWs nCOV^+^ Spn^+^; (c) patients nCOV^+^ Spn^–^; (d) patients nCOV^+^ Spn^+^; and (e) healthy controls. The demographic and clinical characteristics of these 5 groups are shown in Table 1. The selection of HCWs (*n =* 27) and patients with COVID-19 (*n =* 89) for the analysis of immunological parameters was mainly based on (a) the availability of convalescent samples and (b) the pneumococcal carriage status. To assess and compare immune responses to SARS-CoV-2, we analyzed convalescent blood and upper respiratory samples (saliva and nasal lining fluid) in the HCW and patient groups. Access to convalescent samples was restricted in the patient cohorts, as only 39% (100 of 255) of SARS-CoV-2–positive individuals donated samples at the convalescent phase of COVID-19 infection. In both cohorts, nasal and systemic inflammation was also assessed during the acute phase of COVID-19 infection. Samples from healthy adults collected prior to June 2019 were used as healthy controls.

### ELISA for SARS-CoV-2 and influenza virus antigens

An ELISA was performed to quantify the levels of IgG and IgA against SARS-CoV-2 antigens in serum and saliva or nasal lining fluid samples, respectively, whereas IgA, IgA1, and IgA2 were measured in nasal wash samples from LAIV recipients (demographics are shown in Supplemental Tables 1 and 2). LAIV was administered intranasally. Recombinant SARS-CoV-2 RBD and N protein were produced at the Jenner Institute (Oxford, United Kingdom), as reported elsewhere (47). Recombinant S1 and S2 subunits (full-length proteins) were commercially available (Cambridge Bioscience). A seasonal tetravalent-inactivated influenza vaccine (TIV**,** either the 2015/2016 or 2016/2017 formulation) was used as the source of influenza antigens for measuring mucosal IgA and subclasses in nasal washes from the study participants.

The levels of antibodies against SARS-CoV-2 and influenza antigens were quantified, as previously described (17, 48) with minor modifications. Briefly, Nunc 96-well plates were coated with 1 μg/mL SARS-CoV-2 antigen or 0.2 μg/mL TIV and stored at 4°C overnight for at least 16 hours. After coating, plates were washed 3 times with PBS with 0.05% Tween-20 and blocked with 2% BSA in PBS for 1 hour at room temperature. Thawed serum, saliva, nasal fluid, and nasal wash samples diluted in 0.1% BSA-PBS were plated in duplicate and incubated for 2 hours at room temperature alongside an internal positive control (dilution of a convalescent serum) to measure plate-to-plate variation. For the standard curve, pooled sera from SARS-CoV-2–infected participants were used in a 2-fold serial dilution to produce either 8 or 9 standard points (depending on the antigen) that were assigned as AU. Goat anti–human IgG (γ-chain specific, A9544, MilliporeSigma) or IgA (α-chain specific, A9669, MilliporeSigma) or mouse anti–human IgA1 (Fc-specific, ab99794, Abcam) or IgA2 (Fc-specific, ab99800, Abcam) conjugated to alkaline phosphatase was used as a secondary antibody, and plates were developed by adding 4-nitrophenyl phosphate in diethanolamine substrate buffer. ODs were measured using an Omega microplate reader at 405 nm. Blank corrected samples and standard values were plotted using the 4-parameter logistic model (Gen5, version 3.09, BioTek).

### Flow cytometric assays

Cryopreserved PBMCs were used, and all samples were acquired on an Aurora cytometer (Cytek Biosciences) and analyzed with Flowjo software, version 10 (TreeStar).

### Direct ex vivo immune B and T cell phenotyping

#### B cell phenotyping.

PBMCs seeded in 96-well plates were washed (440*g* for 5 min), stained with LIVE/DEAD eFluor 506 Viability Dye for 15 minutes at 4°C (Thermo Fisher Scientific), followed by an extracellular cocktail of monoclonal antibodies, including SARS-CoV-2 S1 and S2 proteins conjugated with biotin-streptavidin for 20 minutes at 4°C, protected from light (Supplemental Table 3). S1 and S2 proteins were conjugated with biotin (EZ Link Conjugation Kit, Thermo Fisher Scientific) and labeled with Streptavidin-BV785 and PE (BioLegend), respectively.

#### T cell phenotyping.

Following stimulation in 96-well U plates, PBMCs were washed (440*g* for 5 min), stained with Viability Dye for 15 minutes at 4^o^C (Thermo Fisher Scientific), followed by an extracellular cocktail of monoclonal antibodies for 20 minutes at 4°C, protected from light (Supplemental Table 4). Cells were washed again (440*g* for 5 min) and then fixed and permeabilized with CytoFix/CytoPerm (BD Biosciences) for 15 minutes at 4^o^C. After the incubation, cells were stained with an intracellular cocktail of monoclonal antibodies (Supplemental Table 4).

### T cell stimulation and intracellular cytokine staining assay

Cells were cultured for 18 hours at 37°C in the presence of SARS-CoV-2–specific peptides (2 μg/mL) in 96-well U-bottomed plates at 1 × 10^6^ PBMCs per well. Overlapping peptides spanning the immunogenic domains of the SARS-CoV-2 spike (Prot_S), nucleocapsid (Prot_N), and S1 subunit (Prot_S1) proteins were purchased from Miltenyi Biotec. GolgiPlug containing brefeldin A and GolgiStop containing monensin (both from BD Biosciences) were added 2 hours after the peptide addition. A stimulation with an equimolar amount of DMSO was performed as a negative control, and staphylococcal enterotoxin B (SEB) (2 μg/mL) was included as a positive control. The following day, cells were harvested from plates and washed and stained for surface markers (Supplemental Table 4).

### Luminex analysis of nasal lining fluid or serum

The Cytokine Human Magnetic 30-Plex panel (Thermo Fisher Scientific) was used to quantitate human nasal and serum cytokines, as previously described (12). Triton-treated nasal fluid and serum were acquired on an LX200 using a 30-Plex Human Magnetic Luminex Cytokine Kit (Thermo Fisher Scientific) and analyzed with xPonent 3.1 software following the manufacturer’s instructions. Samples were run in duplicate, and standards were run on all plates. Calibration and verification beads were run prior to all runs.

### Statistics

Statistical analyses were performed using R software (version 4.0.4) or GraphPad Prism (version 9.0). A 2-tailed statistical test was used throughout the study. Categorical variables were compared using a Fisher’s exact or χ^2^ test. Continuous variables were tested for normality and appropriate statistical tests applied. Non-normally distributed measurements are expressed as the median, and a Mann-Whitney *U* (for 2-group comparisons) or Kruskal-Wallis (for 3- to 5-group comparisons) test was used. Differences were considered significant at a *P* value of less than 0.05, and multiple testing corrections were applied where appropriate. FDR corrections were performed using the Benjamini-Hochberg test at an FDR significance threshold of less than 0.05.

### Study approval

The 2 study protocols were reviewed and approved by the Health Service Research Ethics Committees of the NHS (REF: 20/SC/0147 for the SAFER study and 20/SC/0169 for the FASTER study). The LAIV clinical trial (LAIV/pneumococcus challenge studies) had been previously approved by NHS Research Ethics Committees (14/NW/1460). All participants provided written informed consent and were free to withdraw from the studies at any point.

## Author contributions

EM conceived and designed the study, developed the study protocol and assays, conducted data analysis and interpretation, and wrote the manuscript. JR and BCU developed assays and conducted experiments and data analysis. CS, EN, SP, AH, LH, ELG, KSC, RLB, CTW, and AICA contributed to sample processing and conducted and analyzed experiments. ADHW, SG, MF, KL, HH, AMC, TF and ERA contributed to participant recruitment, sample collection, protocol development, and design of the study. NFW conceived and designed the SAFER study, developed and implemented the SAFER study protocol, performed data analysis, and reviewed and commented on the manuscript. SJD and DP produced and provided reagents and reviewed and commented on the manuscript. RB supervised the study, developed the study protocol, and reviewed and commented on the manuscript. CT interpreted data and reviewed and commented on the manuscript. EB supervised the study, developed the study protocol, interpreted data, and reviewed and commented on the manuscript. LJ, BDG, and DMF conceived and designed the study, developed the study protocol, interpreted data and wrote the manuscript. All authors read and approved the manuscript.

## Supplementary Material

Supplemental data

ICMJE disclosure forms

## Figures and Tables

**Table 1:**
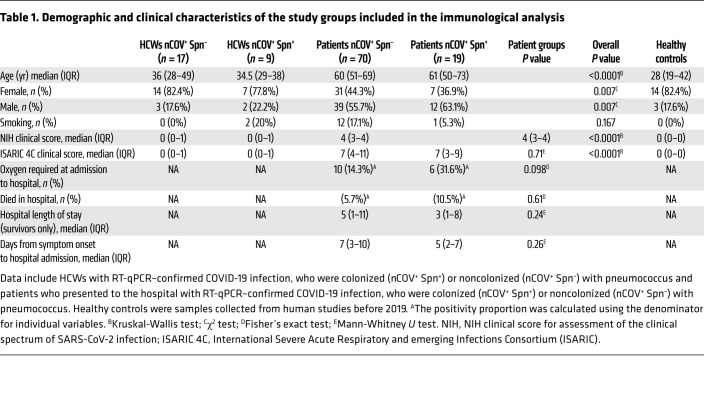
Demographic and clinical characteristics of the study groups included in the immunological analysis

**Table 2:**
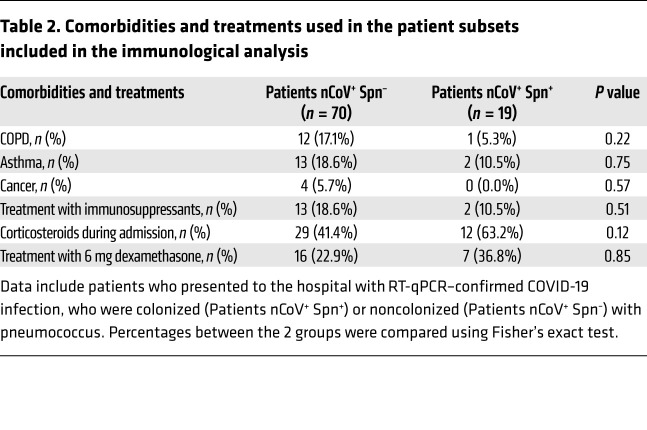
Comorbidities and treatments used in the patient subsets included in the immunological analysis

## Version 1 02/09/2022

In-Press Preview

## Version 2 04/01/2022

Electronic publication
