# Supplementary material for: Streptococcus pneumoniae colonization associates with impaired adaptive immune responses against SARS-CoV-2
Source: J Clin Invest. 2022 Apr 1;132(7):e157124. doi: 10.1172/JCI157124 (PMC8970672; doi:10.1172/JCI157124)
Supplement: Supplemental data [file jci-132-157124-s088.pdf]

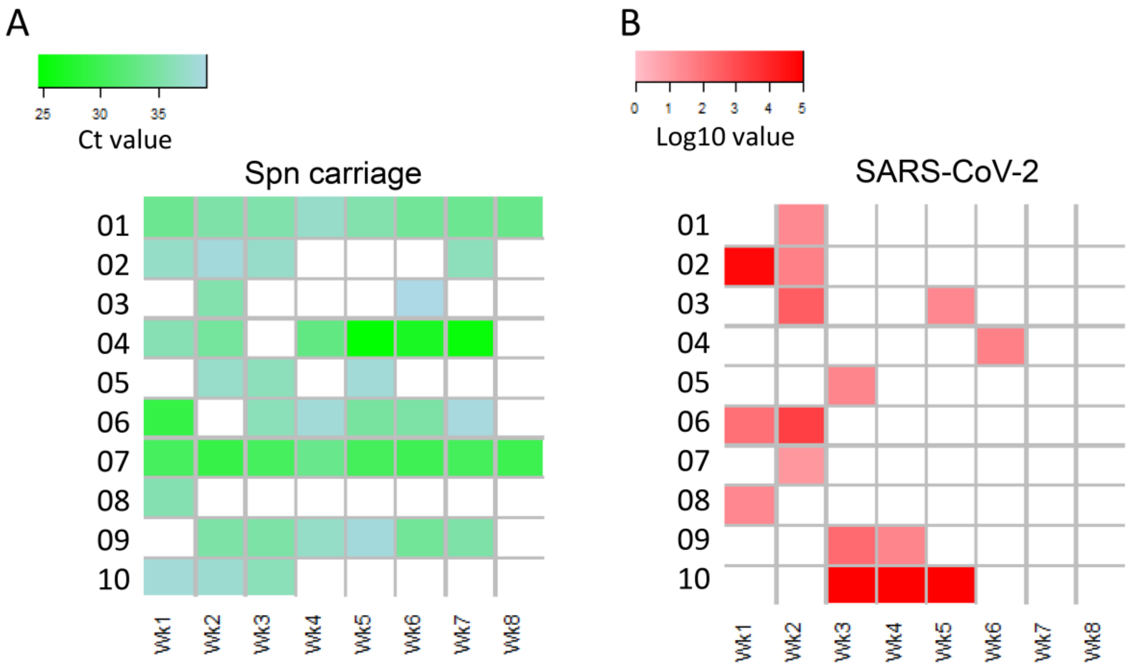

Supplemental figure 1: A) Heatmap showing CT values of pneumococcal DNA detected by *lytA/piaB* qPCR in the nose and throat and/or saliva of Sp (n=10) during the first 8 weeks of enrolment to the study**. B)** Heatmap showing log10 viral load of SARS-CoV-2 detected by qPCR in nose and throat swab and/or saliva of the same participants at week 1 to week 8 of enrolment to the study.

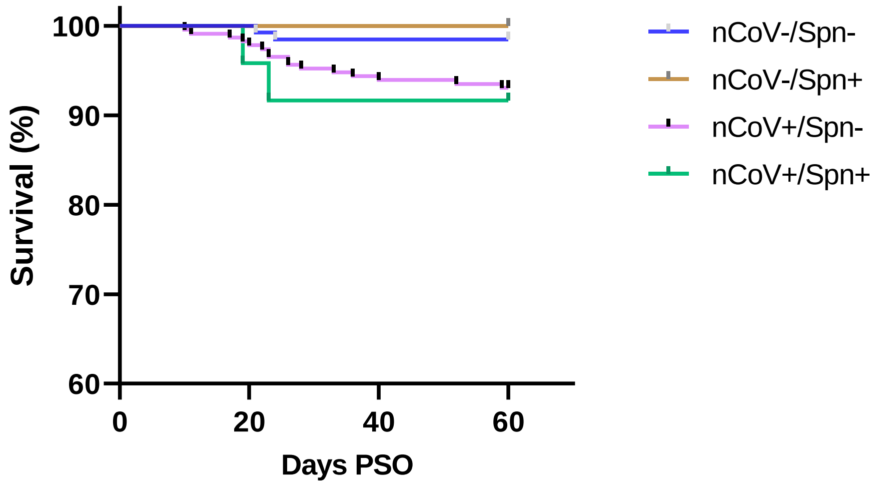

Supplemental figure 2: Percentage of survival rates in patient cohort (n=400), stratified in four groups based on SARS-CoV-2 and pneumococcal carriage status. In patients who represented to hospital with respiratory symptoms but had not confirmed SARS-CoV-2 infection, survival rates were 98.5% (2/134) in Spn- and 100% (11/11) in Spn+ group. In those with COVID-19 disease, survival rates were 93.1% (16/231) in Spn- and 91.7% (2/24) in Spn+ group at observed endpoint during hospitalisation calculated from symptoms onset. Pneumococcal carriage did not associate with decreased survival rates in COVID-19 patients. Comparison of survival curves was performed using log-rank test. There was no statistical difference between the groups (p=0.098).

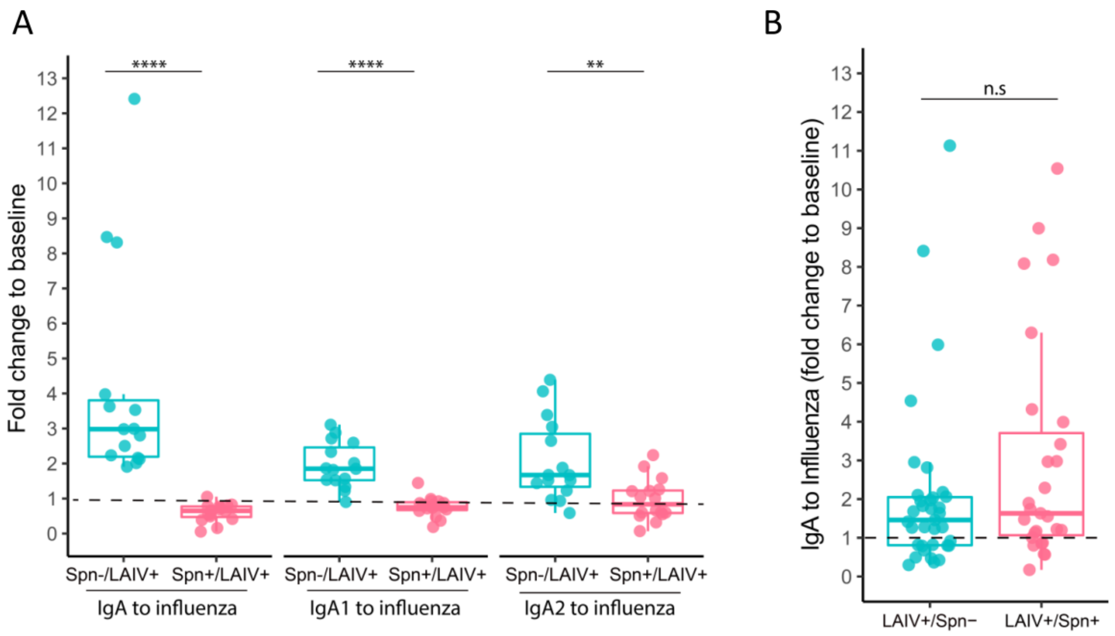

Supplemental figure 3: Mucosal IgA antibodies against influenza antigens. A) Fold change (day 24 post LAIV administration versus baseline) in nasal levels of IgA, IgA1 and IgA2 to influenza antigen stratified by pneumococcal status (Spn-/LAIV+ in light blue, n=15 and Spn+/LAIV+ in light red, n= 16) in individuals challenged with live pneumococcus 3 days before LAIV administration. B) Fold change (day 27 post LAIV administration versus baseline) of nasal IgA to influenza antigens in individuals challenged with live pneumococcus 3 days after LAIV administration (LAIV+/Spn-, n=35 and LAIV+/Spn+, n=27). Medians with IQRs are depicted and each spot represents an individual. **$p<0.01$, ****$p < 0.0001$ by Mann-Whitney test comparing fold-change levels between carriage– and carriage+ subjects.

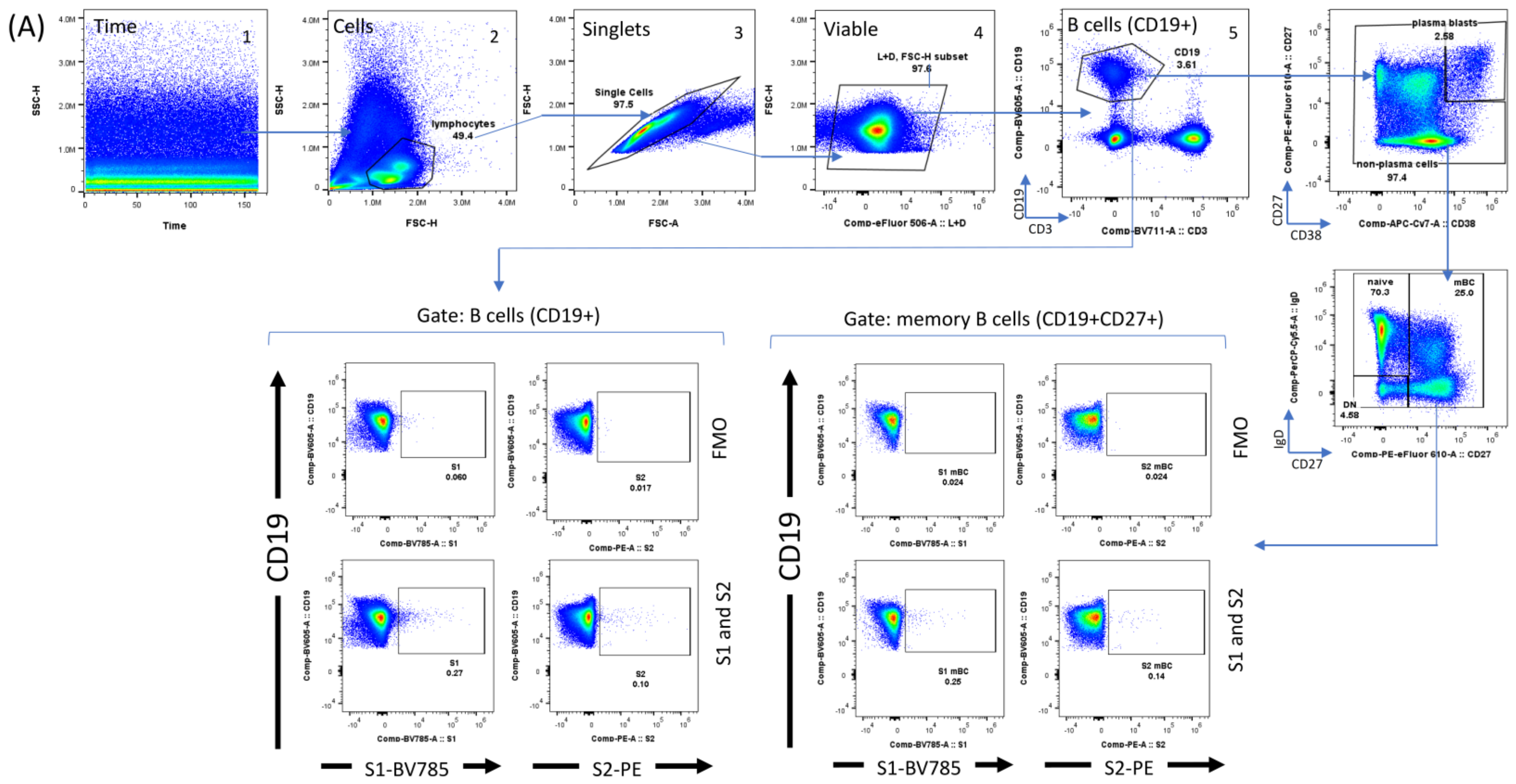

Supplemental figure 4. Gating strategy for B cell subset analysis using a representative PBMC sample. Identification of the B cells based on the following steps from left to right: 1) Time dot-plot to determine the quality of stability of the acquisition 2) Adequate adjustment of the gate of lymphocytes 3) Exclusion of doublets with the identification of singlets improving the accuracy of the analysis 4) Selection of the viable lymphocytes 5) Identification of the B cells based on CD19+ expression. Analysis within B cells (CD19+) subset. Subsequently, we identified plasma blasts (CD27+CD38++) and non-plasma cells (CD38-) based on the expression of CD27 (memory cell marker) vs. CD38 (plasma cell marker). From non-plasma cells we identified the naïve, memory B cells (mBC) and the double negative population based on the expression of IgD vs. CD27. Finally, to delineate the SARS-CoV-2 specific B cells against S1 and S2, we analysed within the B cells (CD19+) and memory B cells (mBC) the percentage of expression S1 and S2 proteins conjugated with biotin and labelled with Streptavidin (BV785 and PE, respectively). Abbreviations: FMO (Fluorescence Minus One).

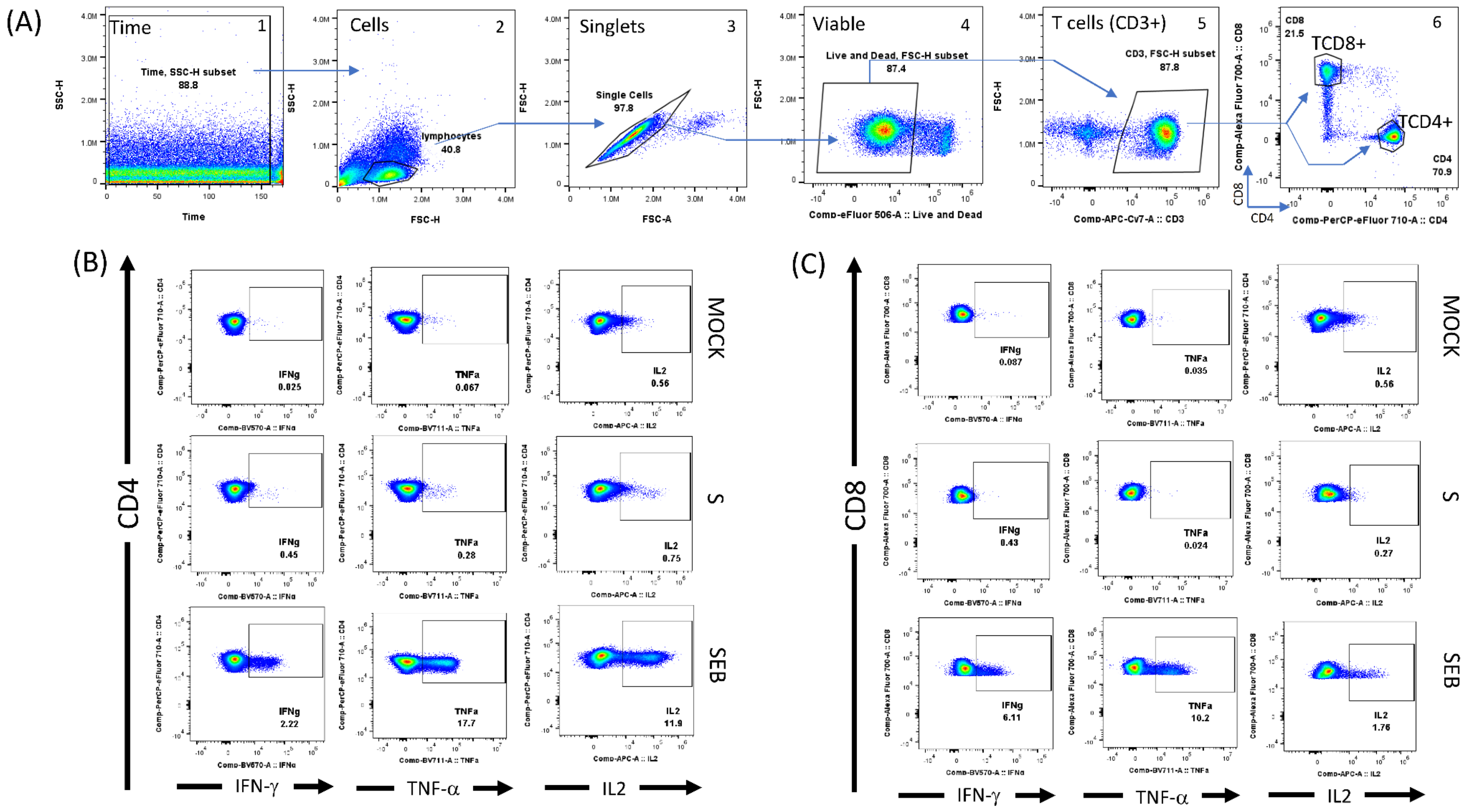

Supplemental figure 5. Gating strategy for T cell subset analysis (CD4+ and CD8+) by flow cytometry using a representative PBMC sample. A) Identification of the T cellular subsets CD4 and CD8 based on the following steps: 1) Time dot -plot to determine the quality of stability of the acquisition 2) Adequate adjustment of the gate of lymphocytes 3) Exclusion of doublets with the identification of singlets improving the accuracy of the analysis 4) Selection of the viable lymphocytes 5) Identification of the T cells based on CD3+ expression 6) Identification of the TCD4+ and TCD8+ within CD3+ cells. **B)** Analysis within TCD4+ cells subset. Representative dot-pots of cytokine production (IFN-γ, TNF-α and IL2) following SARS-CoV-2 Spike (S) protein stimulation (2µg/mL) and SEB (Staphylococcus Enterotoxin B) as positive control compared to mock (unstimulated) within CD4+ cells. TCD4+ cells were also stimulated

SARS-CoV-2 with S1 subunit (S1) and Nucleocapsid (N) (not shown) (C) Analysis within TCD8+ cells subset. Representative dot-pots of cytokine production (IFN-γ, TNF-α and IL2) following SARS-CoV-2 Spike protein stimulation (2µg/mL) and SEB (Staphylococcus Enterotoxin B) as positive control compared to mock (unstimulated) within CD8+ cells. CD8+ cells were also stimulated SARS-CoV-2 with S1 subunit (S1) and Nucleocapsid (N) (not shown).

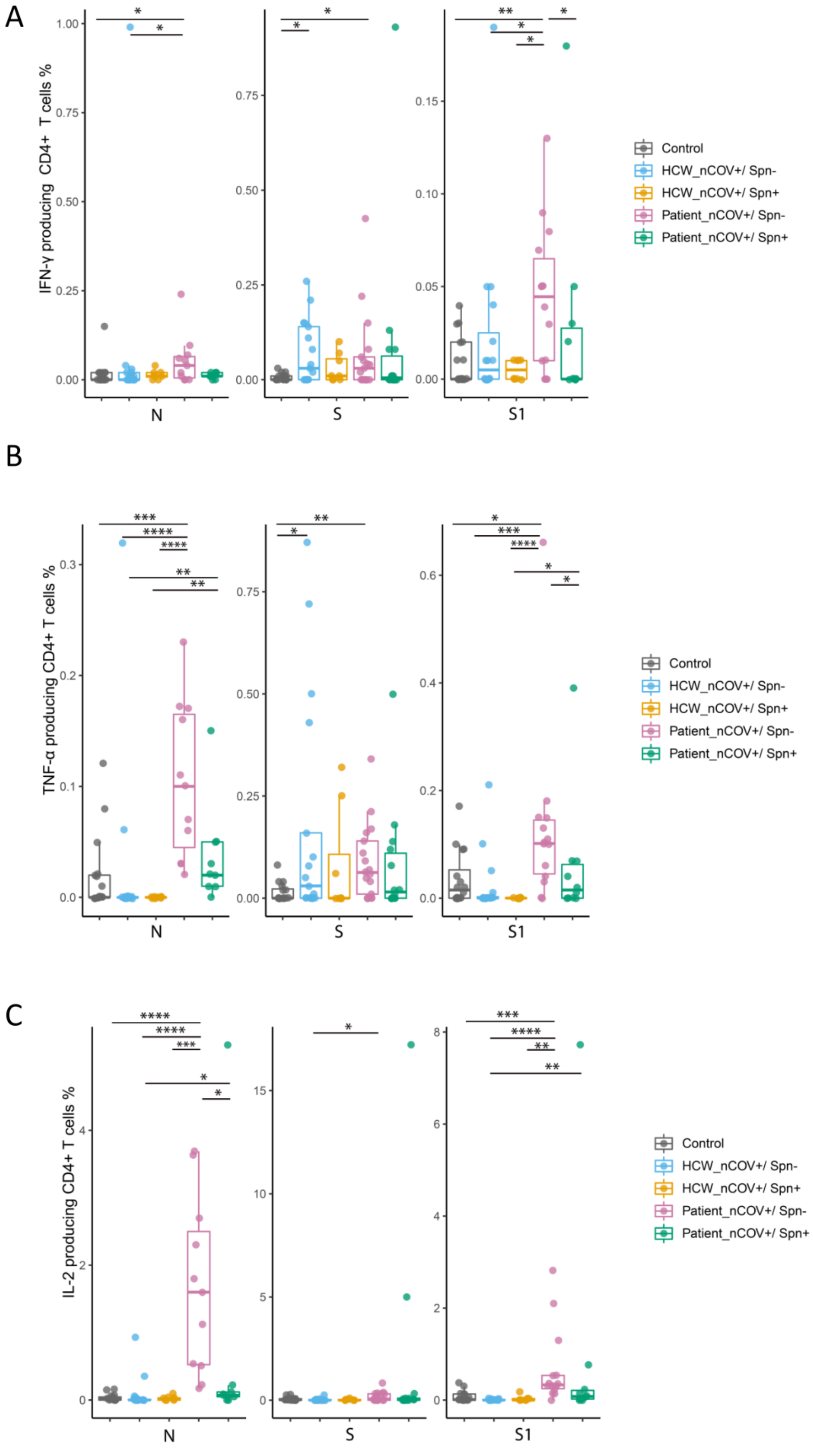
A
IFN-γ producing CD4+ T cells %
N
S
S1
B
TNF-α producing CD4+ T cells %
N
S
S1
C
IL-2 producing CD4+ T cells %
N
S
S1
Control
HCW_nCOV+/ Spn-
HCW_nCOV+/ Spn+
Patient_nCOV+/ Spn-
Patient_nCOV+/ Spn+

Supplemental figure 6. Percentage of A) IFN-γ, B) TNF-α and C) IL-2 producing CD4+ T cells after ex vivo PBMC stimulation with N, S1 and S peptides pools in exposed HCWs (non-colonised; n=17 and Spn-colonised; n=8), recovered patients (non-colonised; n=17 and Spn-colonised; n=14) and healthy control (n=16). One peptide pool was used per condition. SEB was used as a positive control and DMSO as the negative control (non-stimulated cell condition-mock). Background (mock) was subtracted from peptide-stimulated conditions to remove non-specific signal. Medians with IQRs are depicted and each spot represents an individual. *p <0.05, **p<0.01, ***p < 0.001, ****p < 0.0001 by Kruskal-Wallis test.

Supplemental Table 1. Demographics of participants for the concurrent LAIV study.

	**Spn-/LAIV** (n= 15)	**Spn+/LAIV** (n=16)	**Overall** (n=31)
**Demographics**			
Median age (IQR)- yr	20.0 (18.0 -21.0)	20 (19.25 -21.0)	20.0 (19.0 -21.0)
Female- no. (%)	6 (40.0 %)	5 (31.2 %)	11 (35.5 %)

Supplemental Table 2. Demographics of participants for the antecedent LAIV study.

	**LAIV/Spn-** (n= 35)	**LAIV/Spn+** (n=27)	**Overall** (n=62)
**Demographics**			
Median age (IQR)- yr	19.0 (19.0 -21.0)	20.0 (19.0 -22.0)	20.0 (19.0 – 21.0)
Female- no. (%)	22 (62.8 %)	17 (62.9 %)	39 (62.9 %)

Marker	Fluorochrome	Clone	Isotype	Provider	Reference
CD19	BV605	HIB19	Mouse IgG1, κ	Biolegend	302244
CD3	BV711	SK7	Mouse IgG1, κ	Biolegend	344838
CD27	PE-eF610	O323	Mouse IgG1, κ	Thermofisher	61-0279-42
IgD	PerCP-Cy5.5	IA6-2	Mouse IgG2a, κ	Biolegend	348208
CD38	APC-Cy7	HIT2	Mouse IgG1, κ	Biolegend	303534
S1	Strep-BV785	NA	NA	Biolegend	405249
S2	Strep-PE	NA	NA	Biolegend	405203
Live & Dead	e506	NA	NA	Thermofisher	65-0866-14

Supplemental table 3. Summary and specifications of the multiparametric flow cytometry panel used for the B cell analysis by flow cytometry. We developed a multiparametric flow cytometry panel composed with different monoclonal antibodies (from Biolegend and BD Biosciences) including a viability dye (Thermofisher), and SARS-CoV-2 S1 and S2 proteins conjugated with biotin (EZ Link, Thermofisher) and labelled with Streptavidin BV785 and PE (Biolegend), respectively. Electronic compensation was set using CompBeads (BD Biosciences), Arc beads (ThermoFisher) and using the Spectral Flow automated unmixing software (Aurora, Cytek Biosciences) according to manufacturer's instructions. Abbreviations: NA, Not applicable.

Marker	Fluorochrome	Clone	Isotype	Provider	Reference
CD3	APC-Cy7	SK7	Mouse IgG1, κ	Biolegend	344818
CD4	PerCP-e710	SK3	Mouse IgG1, κ	Thermofisher	46-0047-42
CD8	AF700	SK1	Mouse IgG1, κ	Biolegend	344724
Live & Dead	e506	NA	NA	Thermo	65-0866-14
IFN-γ	BV570	4S.B3	Mouse IgG1, κ	Biolegend	502534
TNF-α	BV711	MAb11	Mouse IgG1, κ	Biolegend	502940
IL-2	APC	MQ1-17H12	Rat IgG2a, κ	Biolegend	500307

Supplemental table 4. Summary and specifications of the multiparametric flow cytometry panel used for T cell analysis by flow cytometry. We developed a multiparametric flow cytometry panel composed with extracellular (CD3, CD4 and CD8) and intracellular (cytokines) monoclonal antibodies (from Biolegend and BD Biosciences) including a viability dye (Thermofisher) to assess SARS-CoV-2 specific T cells in HCW and patients with SARS-CoV-2 infection by flow cytometry. Electronic compensation was set using CompBeads (BD Biosciences), Arc beads (ThermoFisher) and using the Spectral Flow automated unmixing software (Aurora, Cytek Biosciences) according to manufacturer's instructions. Abbreviations (NA, Not applicable).

Upper respiratory tract and blood sample collection

HCWs were asked to provide a self-collected flocked combined nasal and throat (NT) swab (Amies, MWE, UK) twice per week. The NT sample were collected by swabbing their throat (tonsil area) first and then a separate swab was used for the nose. Both swabs were combined in the same tube.In addition to two saliva samples (raw material and in 1ml of STGG) into sterile tubes (STARSTED, USA) and two SAM filters (Nasosorption FX, UK) werecollected once per week. Peripheral blood samples were collected by the Clinical Research Unit team. Serum samples were collected on the recruitment day and monthly during the 12weeks follow-up, whereas PBMCs were collected on the day of recruitment and at 3-months visit.

For the patient cohort, recruitment was either in the A&E unit or in wards within 48h of hospital admission. Whole blood, respiratory samples (NT swabs, throat swabs in STGG, saliva in STGG, SAM filters) and urine were collected at the point of patient recruitment. If the patient had a positive SARS-CoV-2 test and stayed in the hospital, then day-2 and a day-7 samples (from study recruitment day)

were collected. All patient participants were invited to visit the Accelerator Research Clinic at Liverpool School of Tropical Medicine and donate samples at the convalescent phase (Day28 ± 25 days from recruitment day). Convalescent patients had to be symptom-free and approximately 3 weeks out from symptoms onset.

For healthy controls, we used samples collected from healthy adults (18-73 years of age) who participated into one of our experimental human pneumococcal carriage studies. These were baseline (prior to inoculation with pneumococcus) blood and respiratory samples, collected between 2015 to 2019 and were considered to be unexposed controls given that SARS-CoV-2 emerged as a novel pathogen in late 2019 (November to December).

PBMC isolation and handling

Whole blood was collected in lithium heparin tubes (BD vacutainer, USA) for all study participants (HCWs, patients and healthy, unexposed donors) and PBMCs were isolated using density-gradient sedimentation in a biosafety level 3 facility. To isolate PBMCs, blood 1:1 diluted in PBS was layered over Ficoll-Paque in a SepMate tube (HCWs and patients) or Leucosep tubes with porous barriers (healthy donors) and centrifuged for 10 min at 1,200*g*. The PBMC layer was collected by quickly pouring the content into a new 50-ml tube. Isolated PBMCs were then cryopreserved in CTL-Cryo ABC media kit (Immunospot, Germany) and frozen overnight at -80°C, and then transferred to liquid nitrogen until further analysis.

Serum isolation and handling

Whole blood was collected in serum separator tubes (SST BD Vacutainer tubes, USA) and centrifuged for 10min at 1200g for serum separation in a biosafety level 3 facility. The serum was then aliquoted and stored at -80°C until further use. Prior to use in assays, serum samples were treated with 1% Triton-X-100 for 1h in room temperature(1).

Respiratory samples handling

NP swabs collected from patients were transferred to LSTM diagnostic laboratory and processed immediately. Self-collected NP swabs from HCWs were stored at -80°C till further use. Throat swabs in STGG, saliva raw and saliva in STGG, SAM filters were stored at -80°C till further use. Where appropriate, respiratory secretion samples were treated with 1% Triton-X-100 for 1h at room temperature prior to use in SARS-CoV-2 ELISAs or cytokine beads assay.

References

1. Patterson EI, Prince T, Anderson ER, Casas-Sanchez A, Smith SL, Cansado-Utrilla C, Turtle L, Hughes GL. Methods of inactivation of SARS-CoV-2 for downstream biological assays. *bioRxiv* 2020.
